# Toward a more personalized motor function rehabilitation in Myotonic dystrophy type 1: The role of neuroplasticity

**DOI:** 10.1371/journal.pone.0178470

**Published:** 2017-05-25

**Authors:** Simona Portaro, Antonino Naro, Antonino Chillura, Luana Billeri, Alessia Bramanti, Placido Bramanti, Carmelo Rodolico, Rocco Salvatore Calabrò

**Affiliations:** 1 Istitute of Scientific Research and Care “IRCCS Centro Neurolesi Bonino-Pulejo”, Messina, Italy; 2 Department of Clinical and Experimental Medicine, University of Messina, Messina, Italy; 3 Institute of Applied Sciences and Intelligent Systems “ISASI Edoardo Caianello”, National Research Council of Italy, Messina, Italy; Nanjing Normal University, CHINA

## Abstract

Myotonic dystrophy type 1 (DM1) is the most prevalent adult muscular dystrophy, often accompanied by impairments in attention, memory, visuospatial and executive functions. Given that DM1 is a multi-system disorder, it requires a multi-disciplinary approach, including effective rehabilitation programs, focusing on the central nervous system neuroplasticity, in order to develop patient-tailored rehabilitative procedures for motor function recovery. Herein, we performed a transcranial magnetic stimulation (TMS) study aimed at investigating central motor conduction time, sensory-motor plasticity, and cortical excitability in 7 genetically defined DM1 patients. As compared to healthy individuals, DM1 patients showed a delayed central motor conduction time and an abnormal sensory-motor plasticity, with no alteration of cortical excitability. These findings may be useful to define patient-tailored motor rehabilitative programs.

## Introduction

Dystrophic myotonias (DMs) are multi-system hereditary diseases with an estimated prevalence of 3–15 per 100000 [1,2], characterized by myotonic symptoms, muscle weakness and atrophy, extra-muscular involvement, cognitive impairment, and dysmorphisms. There are two main DMs forms: Myotonic dystrophy type 1 (DM1), which is the most common adult form of muscular dystrophy (due to a CTG-repeat expansion of at least 50 repeats in the non-coding 3' UTR of the myotonic dystrophy protein kinase(DMPK)-gene) [3], and Myotonic dystrophy type 2 (DM2), which is an autosomal dominant disorder caused by mutations in the zinc finger protein 9(ZNF9)-gene [4]. Within the multisystem involvement, DM1 implies central nervous system abnormalities. In fact, on brain magnetic resonance imaging (MRI), DM1 patients show hyperintense lesions involving the periventricular and deep white matter on T2-weighted sequences, dilated Virchow-Robin spaces [5], and an asymmetric and patchy subcortical white matter involvement [6–8].

Given that DM1 is a multi-system disorder, it requires a multi-disciplinary approach, including effective rehabilitation programs that can help to maintain patient’s quality of life, as well as maximize patient’s physical and psychosocial functions. An effective rehabilitation program can also minimize secondary medical comorbidity, prevent or limit physical deformity, and allow the patient to integrate into society. The study of central nervous system neuroplasticity has significantly contributed to the development of patient-tailored rehabilitative programs for motor function recovery in different neurological conditions [9]. In fact, it is possible to harness the residual plasticity of the brain to maximize functional recovery through, e.g., physical therapy and neuromodulation approaches [9,10]. Therefore, the knowledge of cortical plasticity in DM1 patients could be helpful to personalize the rehabilitative program to the single patient, harnessing the plasticity potential to foster motor recovery, also by implementing neuromodulation strategies [9,11]. Nonetheless, there are few neurophysiological data on the central nervous system available in defined myopathies, including DM1. It has been reported that functional involvement of corticospinal pathway [12,13], subclinical signs of cortical disinhibition [14], disinhibition in the somatosensory cortex [15], elevated central motor thresholds [16], and a deficit of the voluntary movement mechanism [17] can be found in several types of myopathy.

Herein, we performed a transcranial magnetic stimulation (TMS) study aimed at investigating central conduction time, sensory-motor plasticity, and cortical excitability in patients with DM1, in an attempt to find plasticity abnormalities that could be helpful to define patient-tailored motor rehabilitative programs in patients with DM1.

## Materials and methods

### Subjects

Seven patients with a clinical, genetic, and electromyographic evidence of DM1, without clinical or laboratory evidence of other neurological disorders or diseases affecting the peripheral nervous system, without taking drugs modifying muscles and nerve excitability, and with no contraindications to TMS, were consecutively included in the study during the follow-up visits. They were recruited at IRCCS Centro Neurolesi “Bonino-Pulejo” of Messina (Italy) between November 2016 and February 2017. Clinical-demographic characteristics are reported in Table 1. Seven normal individuals were enrolled as a control group (two males, five females, mean age of 24±5 years). The study was reviewed and approved by the Ethics Committee of IRCCS Centro Neurolesi “Bonino-Pulejo” of Messina (Italy) before the study began. The study has been conducted according to the principles expressed in the Declaration of Helsinki. All subjects gave their written informed consent to study participation.

**Table 1:** DM1 clinical-demographic characteristics.

DM1 patients	CTG expansion^a^	Onset	Comorbidities	CRS^b^	MSS^c^
F,49y	E2	25y	H^d^	2	2
F,23y	E2	16y	nasal turbinate stenosis	2	2
M,19y	E2	17y	-	1	3
F,45y	E2	22y	BS1^e^, OSAS^f^	2	3
F,29y	E2	15y	H	2	2
F,17y	E2	15y	-	1	3
F,65y	E2	30y	H, HBV^g^, slight respiratory failure	2	2

^a^ Range of CTG expansion: E1: 20–150; E2: 150–1000; E3: >1000.

^b^ CRS (Clinical rating scale) for Dystrophic Myotonia type 1 (DM1): 1 = presence of myotonia and/or mild functional weakness without functional impairment; 2 = moderate muscle weakness leading to some degree of functional impairment; 3 = muscle weakness with severe functional impairment and in some cases resulting in the subjects being bound to a wheelchair; 4 = bedridden.

^c^ MSS (myotonia severity scale) for DM1 from 0 (absence of myotonia) to 4 (the worst myotonia experienced) [37–39].

^d^ headache

^e^ Brugada syndrome type 1

^f^ Obstructive Sleep Apnea Syndrome

^g^ hepatitis-B

### Experimental sessions

The subjects were seated in a comfortable chair, with both the arms resting on a pillow put over their thighs. Preliminarily, TMS on both primary motor cortices (M1) was carried out using a high-power Magstim 200 stimulator (Magstim Company; Whitland, Dyfed, UK) with a 9 cm circular coil centered over the vertex. The current direction in the coil was counter-clockwise or clockwise for preferential activation of the left and right hemisphere, respectively [18]. We determined the resting motor threshold (RMT) [19] from the left and right relaxed first dorsal interosseous muscle (FDI). Then, ten motor evoked potentials (MEP) were recorded at 120% RMT [20].

To evaluate peripheral motor conduction from the spinal cord to the muscles, we stimulated over the cervical spine. The coil was placed with the lower edge of the C7 spinous process, using a counter-clockwise or clockwise inducing current (as viewed from behind) for the left or right side stimulation, respectively. We measured the latency (from the first deflection of the baseline) of the cortical and cervical MEPs (in ms) and the peak-to-peak amplitude (in mV) of cortical MEPs. The shortest reproducible latency of the responses and the largest peak-to-peak amplitude were selected.

Central motor conduction time (CMCT) from the cortex to C7 was evaluated by the difference of cortical and cervical latencies.

Then, we measured the peak-to-peak MEP amplitude from the right FDI and abductor pollicis brevis muscle (APB) (recording 10 MEPs) during rest and slight tonic contraction at approximately 10–15% of maximum force level. Audiovisual feedback of ongoing EMG activity was provided to ensure a constant strength. The peak-to-peak amplitude of the MEP was measured to characterize corticospinal excitability. In this protocol, MEPs were elicited at rest by using monophasic stimuli at 120% of RMT, delivered through a standard figure-of-eight coil wired to a high-power Magstim 200 stimulator. The active trials were used to measure the duration of the cortically evoked silent period (CSP), which is a marker of long-lasting intracortical inhibition (presumably GABAergic) [21,22]. For CSP measurements, EMG traces were rectified but not averaged. The duration of the CSP was measured in each trial and defined as the time from the onset of the MEP to the reappearance of sustained EMG activity [23].

Finally, we assessed sensory-motor plasticity using a rapid paired associative stimulation (rPAS) paradigm. rPAS consisted of 600 pairs delivered to the left M1 at a rate of 5Hz for 2 min.

Each pair of stimuli consisted of an electrical conditioning stimulus given to the right ulnar nerve at twice the sensory threshold followed, after 25ms, by a biphasic transcranial magnetic stimulus (at 90% of active motor threshold -AMT) given to the left M1 by using a Magstim repetitive stimulator wired to a figure-of-eight coil [24]. In this protocol, MEPs were recorded from both FDI and abductor pollicis brevis muscle (APB) of the right hand (using monophasic stimuli delivered through a standard figure-of-eight coil wired to a high-power Magstim 200 stimulator) before (T0) and after (immediately, T1, 30-minute, T2, and 60-minute, T3) the end of rPAS.

### Data recording and analysis

EMG signals were recorded with Ag–AgCl surface electrodes from the muscles using a belly-tendon montage. Signals were amplified and bandpass filtered (10Hz to 1KHz) by a Digitimer D-150 amplifier and stored at a sampling rate of 10 kHz on a personal computer for off-line analysis (Signal Software; Cambridge Electronic Design, Cambridge, UK).

The baseline neurophysiological data were compared using Student’s *t*-test; clinical-electrophysiological correlations were tested using a Spearman correlation. According to previous work, patient’s values were considered abnormal when they were beyond ±2SD of the normal mean [12]. The effects of rPAS on peak-to-peak MEP amplitude were evaluated by two-way repeated measures ANOVA with the factors *time* (four levels: T0, T1, T2, and T3) and *group* (two levels: DM1 and HC). The factor *muscle* (two levels: APB and FDI) was added to assess the topographic specificity of the rPAS aftereffects. The Greenhouse-Geisser method was used if necessary to correct for non-sphericity. Conditional on a significant *F* value, *post-hoc* paired-sample *t*-tests were performed to explore the strength of main effects and the patterns of interaction between experimental factors. A Bonferroni corrected p-value of *<*0.05 was considered significant. All data are given as mean±SD.

## Results

All the recruited patients completed the experimental procedure, without reporting any adverse effects during or after the experiments.

**Fig 1:**
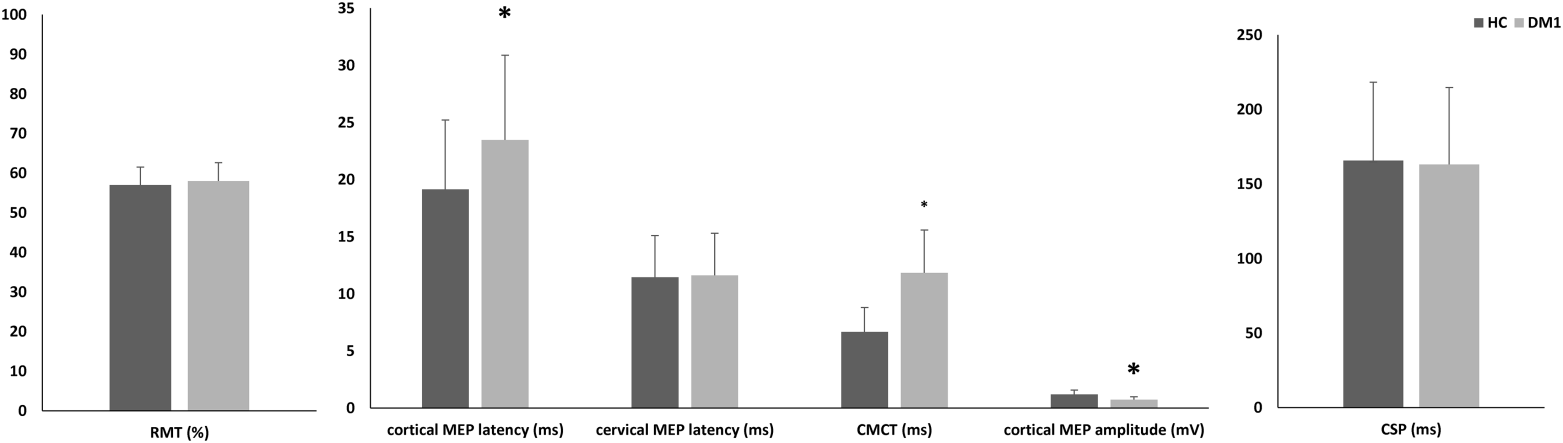
Cortical excitability and central motor conduction time. RMT resting motor threshold; MEP motor evoked potential; CMCT central motor conduction time; CSP cortical silent period; HC healthy control; DM1 myotonic dystrophy type 1.

Fig 1 Illustrates the baseline TMS findings on RMT, cortical and cervical MEP, and CSP. As compared to HC, cortical MEP latency was delayed in latency (p = 0.002), smaller in amplitude (p<0.001), and largely abnormal in morphology (polyphasic and desynchronized responses). There were no significant differences concerning cervical MEP latency, amplitude, and morphology, CSP and RMT (each p = 0.4). Consequently, CMCT was increased in DM1 patients (p<0.001).

**Fig 2:**
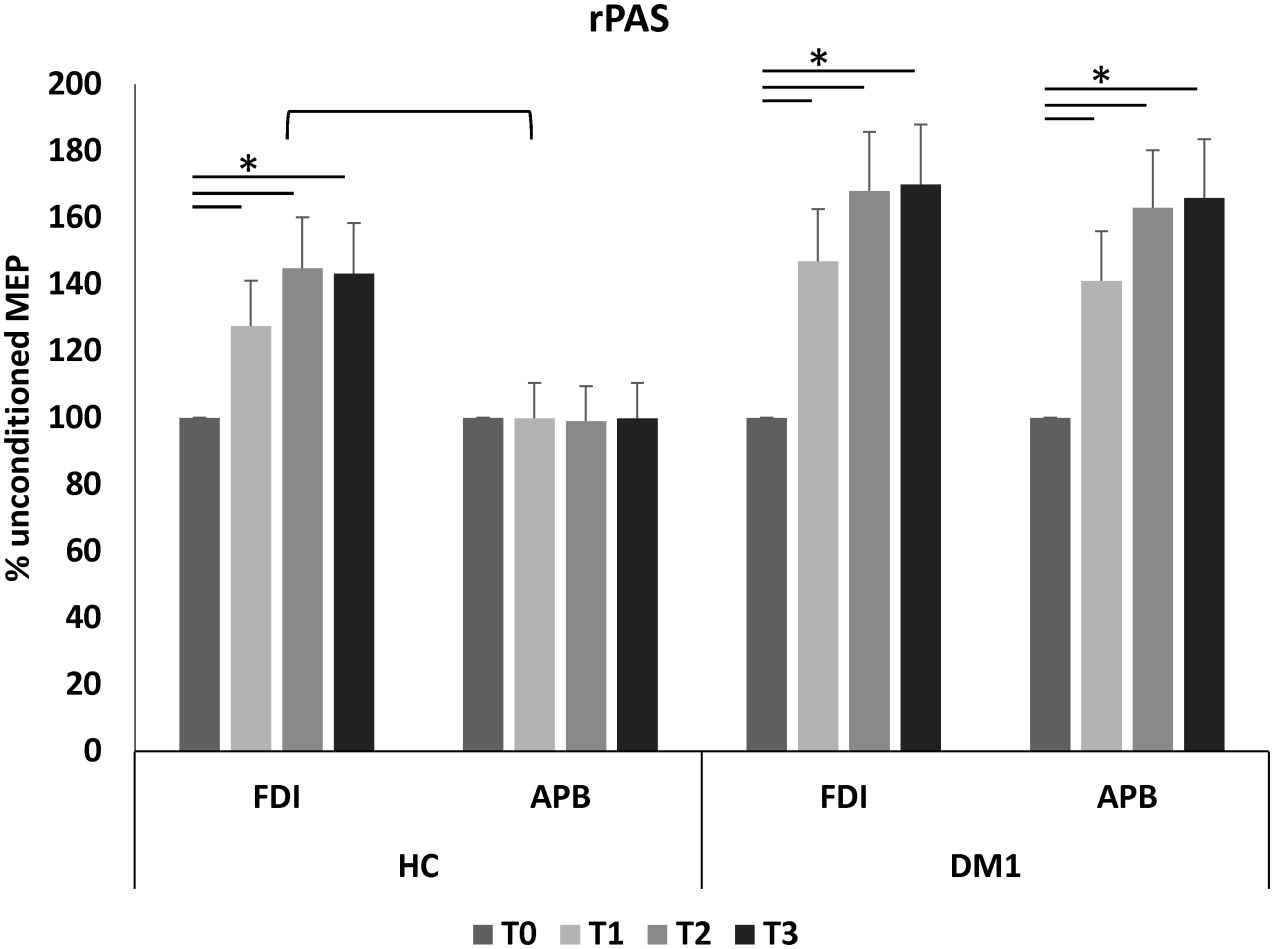
Repetitive paired associative stimulation aftereffects. FDI first dorsal interosseous; APB abductor pollicis brevis; HC healthy control; DM1 myotonic dystrophy type 1; rPAS repetitive paired associative stimulation; * indicates a significant change of MEP amplitude from baseline (T0). Square bracket indicates the topographic specificity of rPAS aftereffects only in HC.

**Fig 3:**
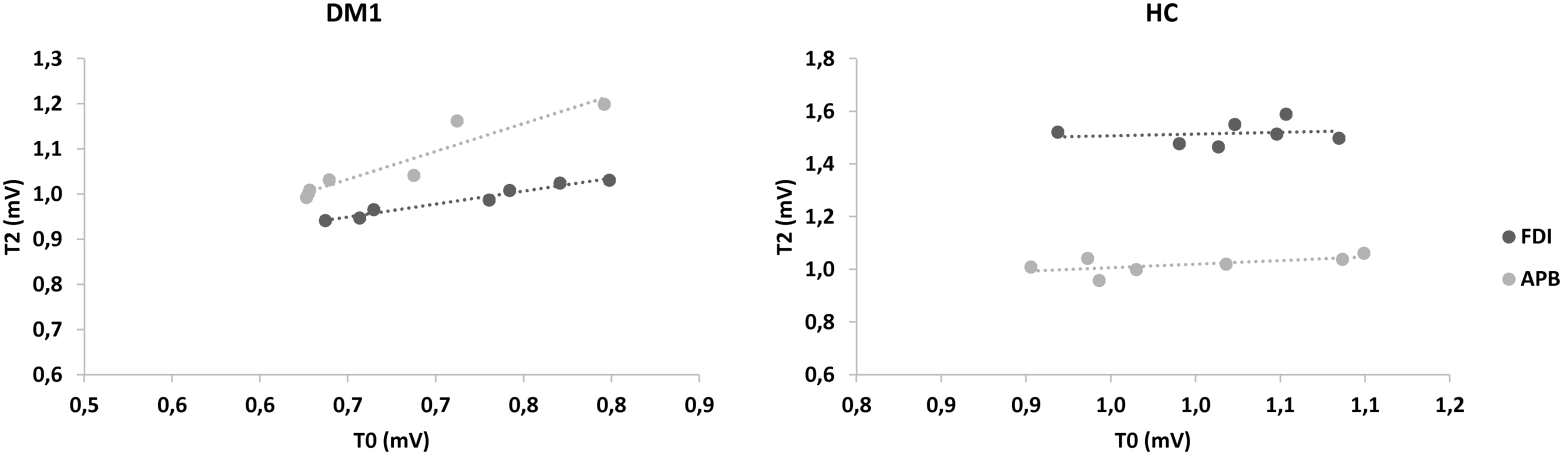
Scatterplots of the correlation between motor evoked potential at baseline and repetitive paired associative stimulation aftereffects at T2. FDI first dorsal interosseous; APB abductor pollicis brevis; HC healthy control; DM1 myotonic dystrophy type 1.

Noteworthy, we found significant rPAS aftereffects (*time*×*group*×*muscle* F = 2.4, p = 0.01), which consisted of a MEP amplitude increase in both the muscles tested in DM1 patients (*time*×*muscle* F = 0.4, p = 0.8; *time* effect F = 84, p<0.001), up to T3 (each *post-hoc t*-test p<0.001) (Fig 2), whilst a preserved topographic specificity (i.e., MEP amplitude increase limited to the FDI muscle) was found in HC (F = 99, p<0.001) up to T3 (each *post-hoc t*-test p<0.001) (Fig 2). Further, we found that the increase of rPAS aftereffects in both the muscles tested at T2 (in which we had the most relevant amplitude increase) in DM1 individuals were both correlated with baseline MEP amplitude (APB r = 0.953, p = 0.001; FDI r = 0.894, p = 0.007), differently from HC individuals (both muscles p = 0.9) (Fig 3). No correlations were found between the electrophysiological parameters and the clinical-demographic characteristics. The dataset is provided in S1 Table.

## Discussion

The novelty of our data consists of the demonstration of wide abnormalities of sensorimotor plasticity in DM1 individuals, beyond corticospinal tract abnormalities (i.e., MEP amplitude reduction and CMCT increase). It has been proposed that MEP alterations may result from muscular atrophy [25], whereas the increase of CMCT may depend on a subtle lesion in the corticospinal tract, accounting for either a reduction in the number of descending volleys or a temporal dispersion of the descending volleys (that could determine a longer time to achieve the temporal summation necessary for the activation of spinal motorneurons) [12]. In addition, DM1 patients show reduced connectivity in large frontoparietal networks, frontal white matter alterations [6–8], and increased parietal-cerebellar connectivity, which have been interpreted as compensatory reorganization phenomena [26]. In keeping with these issues, the abnormally increased sensorimotor plasticity in DM1 we found may be interpreted as a compensatory phenomenon to maintain a sufficient corticospinal output [27]. In fact, the lack of topographic specificity and the increase of rPAS aftereffects were both correlated with baseline MEP amplitude, differently from HC individuals [24], and there were no abnormalities of RMT and CSP. About that, the mutation of DMPK-gene may involve primarily cell shape determination, the regulation of actin-myosin contractility, and the nuclear envelope stability rather than the modulation of the activity of voltage-gated ion channels [28]. These may altogether determine a more structurally involvement of brain cell functions, thus justifying corticospinal tract alterations rather than cell membrane dis-excitability.

Alternatively, rPAS abnormality may reflect a use-dependent plasticity [29–32], implying that myopathic patients perform greater efforts for various activities of daily living, thus explaining the easy fatigability of such individuals. Furthermore, muscular dystrophy may itself change sensory feedback from muscle spindles, which could modify sensorimotor plasticity [12,33]. Hence, rPAS abnormalities may also be considered as an additional epiphenomenon of a multisystem syndrome as the DM1 is.

It should be further investigated whether central nervous system alterations may yield to more severe DM1 symptoms. Indeed, we did not find any correlation between neurophysiological parameters and the degree of clinical impairment, as observed by other authors [34]. Hence, the severity of the electrophysiological changes does not correlate with the degree of muscular atrophy and weakness in DM1. Such data suggests that the central nervous system motor dysfunctions are independent of the primitive muscle damage and are part of the manifestations of the disease. However, these data need to be confirmed in larger sample size studies.

Thanks to the advances in technical supports, such as electrical wheelchairs, home ventilation, and canes, rehabilitation management of these patients went from maintaining walking abilities as long as possible (with physiotherapy, stretching, and braces) to improving upper limb function, taking into account that many technical and electronic supports require a certain ability of the upper limbs [9]. The knowledge and the possibility to shape cortical plasticity to improve upper limb motor function by using conventional and non-conventional (e.g. neurorobotics and non-invasive neuromodulation) protocols arose great interest in neurorehabilitation [9,35]. Therefore, our data could be used to design patient-tailored rehabilitation program and identify the patients who may benefit from neuromodulation approach, in an attempt to restore the abnormal cortical plasticity related to DM1 and improve neurorehabilitation outcomes. Moreover, the possibility to shape neuroplasticity in patients with DM1 may be of notable importance, given that a maladaptive plasticity can worsen the clinical phenotype and the motor recovery, and make the patient more disabled [36].

In conclusion, our findings suggest that patients with DM1 present an involvement of motor descending pathways and cortical sensory-motor plasticity that are independent from primitive muscle damage (i.e., they belong to the wide spectrum of the DM1 multisystem dysfunctions). Overall, such findings should be taken into account to manage these patients better and to establish a more individualized and functional therapeutic approach, as an abnormal plasticity may negatively affect the motor function rehabilitation outcome. Indeed, the knowledge of cortical plasticity in DM1 patients could be helpful to predict the motor outcome, to follow patient’s rehabilitation progress, and to harness the residual plasticity in order to foster motor recovery by implementing neuromodulation strategies.

## Supporting information

S1 Table Supplementary table.xlsx contains the dataset of the study. (XLSX)
